# Crossed Cerebellar Diaschisis in Patients with Diffuse Glioma Is Associated with Impaired Supratentorial Cerebrovascular Reactivity and Worse Clinical Outcome

**DOI:** 10.1007/s12311-020-01174-y

**Published:** 2020-07-31

**Authors:** Martina Sebök, Christiaan Hendrik Bas van Niftrik, Matthias Halter, Aimee Hiller, Katharina Seystahl, Athina Pangalu, Michael Weller, Christoph Stippich, Luca Regli, Jorn Fierstra

**Affiliations:** 1 Department of Neurosurgery, University Hospital Zurich, University of Zurich, Frauenklinikstrasse 10, 8091 Zurich, Switzerland; 2 Clinical Neuroscience Center, University Hospital Zurich, University of Zurich, Zurich, Switzerland; 3 Department of Neurology, University Hospital Zurich, University of Zurich, Zurich, Switzerland; 4 Department of Neuroradiology, University Hospital Zurich, University of Zurich, Zurich, Switzerland

**Keywords:** BOLD fMRI, Cerebrovascular reactivity, Supratentorial hemodynamic, Crossed cerebellar diaschisis, Diffuse glioma

## Abstract

Crossed cerebellar diaschisis (CCD) can be associated with impaired cerebrovascular reactivity (CVR) and poor clinical outcome, but whether this holds true for patients with diffuse glioma is unknown. With blood oxygenation level–dependent (BOLD)-CVR imaging, we determined the presence of CCD in patients with diffuse glioma and investigated its relationship with cerebrovascular reactivity and clinical outcome. For eighteen enrolled subjects (nineteen datasets) with diffuse glioma, CCD was deferred from BOLD-CVR using a predetermined cerebellar asymmetry index (CAI) cutoff value of 6.0%. A FET-PET study was done as a verification of the CCD diagnosis. BOLD-CVR values as well as clinical performance scores (i.e., Karnofsky performance score (KPS), disability rating scale (DRS), and modified Rankin scale (mRS)) by BOLD-CVR scan at 3-month clinical follow-up were assessed and compared for the CCD-positive and CCD-negative group. CCD was present in 26.3% of subjects and strongly associated with impaired BOLD-CVR of the affected (i.e., the hemisphere harboring the glioma) and unaffected supratentorial hemisphere (CCD(+) vs. CCD(−): 0.08 ± 0.11 vs. 0.18 ± 0.04; *p* = 0.007 and 0.08 ± 0.12 vs. 0.19 ± 0.04; *p* = 0.007, respectively). This finding was independent of tumor volume (*p* = 0.48). Furthermore, poorer initial (by scan) clinical performance scores at follow-up were found for the CCD(+) group. The presence of crossed cerebellar diaschisis in patients with diffuse glioma is associated with impaired supratentorial cerebrovascular reactivity and worse clinical outcome.

**Electronic supplementary material:**

The online version of this article (10.1007/s12311-020-01174-y) contains supplementary material, which is available to authorized users.

## Introduction

In patients with diffuse glioma, crossed cerebellar diaschisis (CCD)—a depression of cerebral blood flow and metabolism in the cerebellar hemisphere contralateral to the supratentorial tumor—may potentially have important clinical and prognostic implications [1].

Previous investigations of diffuse glioma and CCD have focused on structural parameters like tumor location, tumor size, and presence of edema but lacked a comprehensive hemodynamic investigation, despite the fact that impaired cerebrovascular reactivity (CVR) may play a major role in CCD pathophysiology and clinical outcome [2–4].

Blood oxygenation level–dependent fMRI (BOLD)-CVR with a standardized carbon dioxide (CO_2_) stimulus [5, 6] is a contemporary technique for investigating CVR impairment in patients with diffuse glioma [7, 8]. It has also shown its utility in accurately detecting CCD, albeit this has been mostly investigated in cerebrovascular patients [3, 9].

With BOLD-CVR and O-(2-[18F]fluoroethyl)-L-tyrosine positron emission tomography (FET-PET) imaging, we determined the presence of CCD in patients with diffuse glioma and investigated the relationship of CCD with cerebrovascular reactivity and clinical outcome.

## Materials and Methods

At the University Hospital Zurich, patients with diffuse glioma according to the 2016 WHO classification [10] were selected from a prospective “BOLD-CVR glioma database” based on the following inclusion criteria: subjects with age > 18 years that underwent a clinical metabolic FET-PET and BOLD-CVR imaging within 6 weeks. The diagnosis of diffuse glioma was confirmed with histopathological analysis for primary gliomas and, where available, for recurrent gliomas. For the remaining recurrent gliomas, the diagnosis was done based on neuroimaging RANO criteria [11] and confirmed by interdisciplinary neuro-oncological tumor board.

All the subjects signed an informed consent before they underwent a BOLD-CVR study. The Cantonal Ethics Committee of the Canton Zurich, Switzerland (KEK-ZH-Nr. 2012-0427), approved this study. The study was performed in accordance with the ethical standards as laid down in the 1964 Declaration of Helsinki and its later amendments. Subjects with cerebellar vascular or oncological pathologies or subjects with medical (oncological chemotherapy), radiotherapeutic, or surgical interventions between the BOLD-CVR and FET-PET examination were excluded from further analysis.

### Image Acquisition and Processing

#### BOLD-CVR Determination

BOLD data and high-resolution T1 imaging were acquired on a 3-tesla Skyra VD13 (Siemens Healthcare, Erlangen, Germany) with a 32-channel head coil. The imaging protocol for BOLD and a three-dimensional (3D) T1-weighted magnetization prepared rapid acquisition gradient echo (MPRAGE) image was performed as published [3].

During the BOLD sequence, the carbon dioxide stimulus was modulated by a computer-controlled gas blender with prospective gas targeting algorithms (RespirAct, Thornhill Research Institute, Toronto, Canada) [12]. The RespirAct allows for precise targeting of arterial partial pressure of oxygen and CO_2_. All subjects were initially maintained on their resting CO_2_ baseline. The BOLD-CVR protocol consists of clamping the subject on its initial resting baseline breathing for approximately 100 s and then inducing a hypercapnia (~ 10 mmHg above resting CO_2_ baseline) for 80 s and a second 100 s of resting baseline [3, 13].

All the acquired BOLD volumes were transferred to an external computer and pre-processed with SPM 12 (Statistical Parameter Mapping Software, Wellcome Department of Imaging Neuroscience, University College of London, London, UK). The BOLD fMRI volumes were pre-processed using time and motion correction and normalized into Montreal Neurological Institute space. Last, a smoothing using a Gaussian Kernel of 6 was used.

To determine the BOLD-CVR, first a voxel-wise temporal shifting for optimal physiological correlation of the BOLD signal and CO_2_ time series was performed. Then, the BOLD-CVR, defined as the percentage BOLD fMRI signal change/mmHg CO_2_, was calculated from the slope of a linear least square fit of the BOLD signal time course to the CO_2_ time course over the range of the whole protocol. Extra BOLD fMRI volumes (40 volumes) were acquired to allow for potential temporal shift. A more extensive description of the BOLD-CVR calculation pipeline can be found in our previous work [5].

### Crossed Cerebellar Diaschisis Determination

#### BOLD-CVR Cerebellar Asymmetry Index Calculation to Diagnose Crossed Cerebellar Diaschisis

For the BOLD-CVR volumes, a cerebellar asymmetry index (CAI) (ipsilateral hemisphere–contralateral *crossed* hemisphere)/ipsilateral hemisphere * 100) was determined using a predefined cerebellar mask. Ipsilateral was defined as the side of the affected supratentorial hemisphere (i.e., the hemisphere harboring the glioma lesion). Derived from our previous study, CCD was diagnosed with a cutoff value of 6.0% CAI [3].

#### Corroborating FET-PET Examination to Verify the BOLD-CVR-Based Crossed Cerebellar Diaschisis Diagnosis

Historically, PET examinations are considered the clinical reference standard to diagnose CCD. Here, the presence of hypometabolism in the affected cerebellar hemisphere is considered pathognomonic for CCD [14–16]. For this study, FET-PET scans were performed according to our clinical standard on an ECAT EXACT HRþ scanner (Siemens Healthcare, Erlangen, Germany). The scanner acquired 63 contiguous transaxial planes, simultaneously covering 15.5 cm of axial field of view. After a 15-min transmission scan (germanium-68 sources), a target dose of 185 MBq of FET was injected intravenously. PET acquisition in 3-dimensional mode was started 30–40 min after injection (128 _ 128 matrix). Data were reconstructed by filtered back projection using a Hann filter after correction for scatter and attenuation.

In a similar fashion as for the BOLD fMRI images, a CAI was determined for the FET-PET images. To allow for an optimal normalization of the PET images to MNI space, the PET images were first coregistered to the mean BOLD fMRI image, which was created during motion correction of the BOLD fMRI images. The normalization to MNI space was then done with the same SPM algorithm as for the BOLD fMRI images.

#### Volume of Interest Determination

Three-dimensional tumor masks were determined and manually drawn from the current state-of-the-art MRI protocol by a neuroradiologist with > 20 years of experience (A.P.) using the iPlan software (BrainLab AG, Munich, Germany). The tumor borders were drawn on every slice where the tumor was visible on the T1-weighted contrast-enhanced scans or on every slice of FLAIR and/or T2 sequences in the case of absent contrast-enhanced lesion, obtaining a 3-dimensional spherical volume of interest. These tumor masks were overlaid on the BOLD-CVR maps to obtain mean intralesional values in those specific regions.

### Assessment of Clinical Performance Status and Neurological Outcome

The Karnofsky performance status (KPS) and disability rating scale (DRS) were used to evaluate the performance status at the moment of scan for glioma or recurrent tumor and at 3-month follow-up. To assess the neurological status initially and at 3-month follow-up, modified Rankin scale (mRS) values were determined.

### Statistical Analysis

We performed the statistical analysis using the SPSS Statistics 26 (IBM Corp., Armonk, NY). All normally distributed continuous variables are reported as mean ± standard deviation (SD). Categorical ordinal variables are presented as median (interquartile range), whereas dichotomous variables are shown as frequency (%). Means of normally distributed continuous variables from the CCD-positive and CCD-negative groups were compared by an independent Student’s 2-tailed *t* test, where *p* < 0.05 was considered statistically significant. ANCOVA was used to statistically control the effect of covariates (WHO grade and primary/recurrent tumor) for BOLD-CVR findings between CCD(+) and CCD(−) groups. Tumor volume was used as covariate to correct the impact on clinical outcome and performance status between CCD(+) and CCD(−) groups. To identify a relationship between the whole cohort BOLD-CVR CAI, whole-brain CVR, and clinical status by BOLD-CVR scan as well as the outcome after 3 months using KPS, DRS, and mRS, we used a Spearman rank-order correlation analysis with partial correction for tumor volume.

## Results

### Study Population Characteristics

**Fig. 1:**
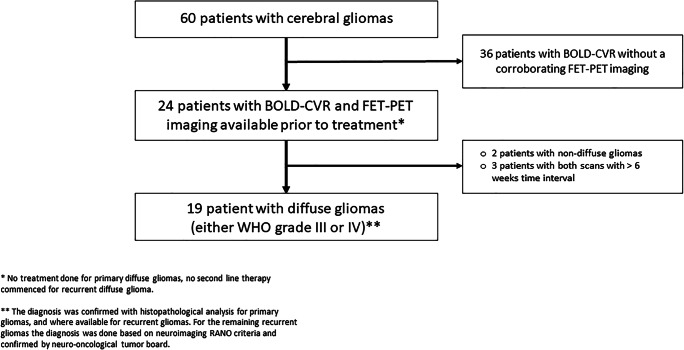
Study flowchart. From the prospective “BOLD-CVR database,” we extracted 60 patients with glioma who underwent BOLD-CVR imaging at first diagnosis of tumor or at diagnosis of tumor recurrence. From 60 glioma patients, 24 patients underwent BOLD and FET-PET imaging prior to treatment (no treatment (i.e., no surgery) done for primary diffuse gliomas, no second line therapy initiated for recurrent diffuse glioma) in the time frame of 6 weeks. Two patients with non-diffuse gliomas and three patients with > 6 weeks interval between BOLD-CVR and PET scans were excluded from the study. Eighteen patients with 19 datasets (one patient underwent the protocol twice—initial and by tumor recurrence) with diffuse glioma (either IDH mutant WHO grade III or IDH wildtype WHO grade IV) were eligible for further analysis (one patient underwent the protocol twice—initial and by tumor recurrence). The diagnosis was confirmed with histopathological analysis for primary gliomas and, where available, for recurrent gliomas BOLD, blood oxygenation level–dependent; CVR, cerebrovascular reactivity, defined as percentage BOLD signal change per mmHg CO_2_; FET-PET = O-(2-[18F]fluoroethyl)-L-tyrosine positron emission tomography; WHO, World Health Organization

A flowchart illustrating patient inclusion can be found in Fig. 1. The 18 included diffuse glioma patients had the following specific diagnoses: primary or recurrent IDH mutant, 1p/19-codeleted anaplastic oligodendroglioma (*n* = 9), IDH mutant anaplastic astrocytoma (*n* = 2), or IDH wildtype glioblastoma (*n* = 7). The mean age was 52.4 ± 12.3 years, and 84% of the subjects were male. Relevant clinical and baseline characteristics of the study population are presented in Table 1 and in the supplemental file (Table 1). No significant differences between CCD(+) and CCD(−) subjects were seen in primary/recurrence tumor, tumor location, histological grade, and molecular analysis (Table 1).

**Table 1:** CCD according to BOLD-CVR: relevant clinical and baseline characteristics

	Total cohort (*n* = 19)	Crossed cerebellar diaschisis positive group (*n* = 5)	Crossed cerebellar diaschisis negative group (*n* = 14)	*p*-value
Age (mean ± SD)	52.4 ± 12.3	57.5 ± 11.5	50.6 ± 12.4	0.30
Male	16 (84.2)	5 (100)	11 (78.6)	0.29
Smoking	4 (21.1)	0	3 (21.4)	0.95
Primary	7 (36.8)	3 (60)	4 (28.6)	0.23
Right hemispheric	11 (57.9)	3 (60)	7 (50)	0.92
Frontal lobe tumor	10 (52.6)	2 (40)	7 (50)	0.54
WHO grade IV	7 (36.8)	3 (60)	4 (28.6)	0.23
MGMT promotor methylated	4 (21.1)	1 (20)	3 (21.4)	0.99
IDH1 mutation	12 (63.2)	2 (40)	10 (71.4)	0.23
ATRX loss	7 (36.8)	2 (40)	5 (35.7)	0.95
1p/19q deletion	10 (52.6)	1 (20)	9 (64.3)	0.51

All data are presented as *n* (%) unless stated otherwise. *ATRX* α thalassemia/mental retardation syndrome X-linked, *IDH* isocitrate dehydrogenase, *LOH* loss of heterozygosity, *MGMT* O^6^-methylguanine-DNA methyl-transferase, *n* number, *SD* standard deviation, *WHO* World Health Organization

### CCD Determination with BOLD-CVR and Verification with FET-PET

**Fig. 2:**
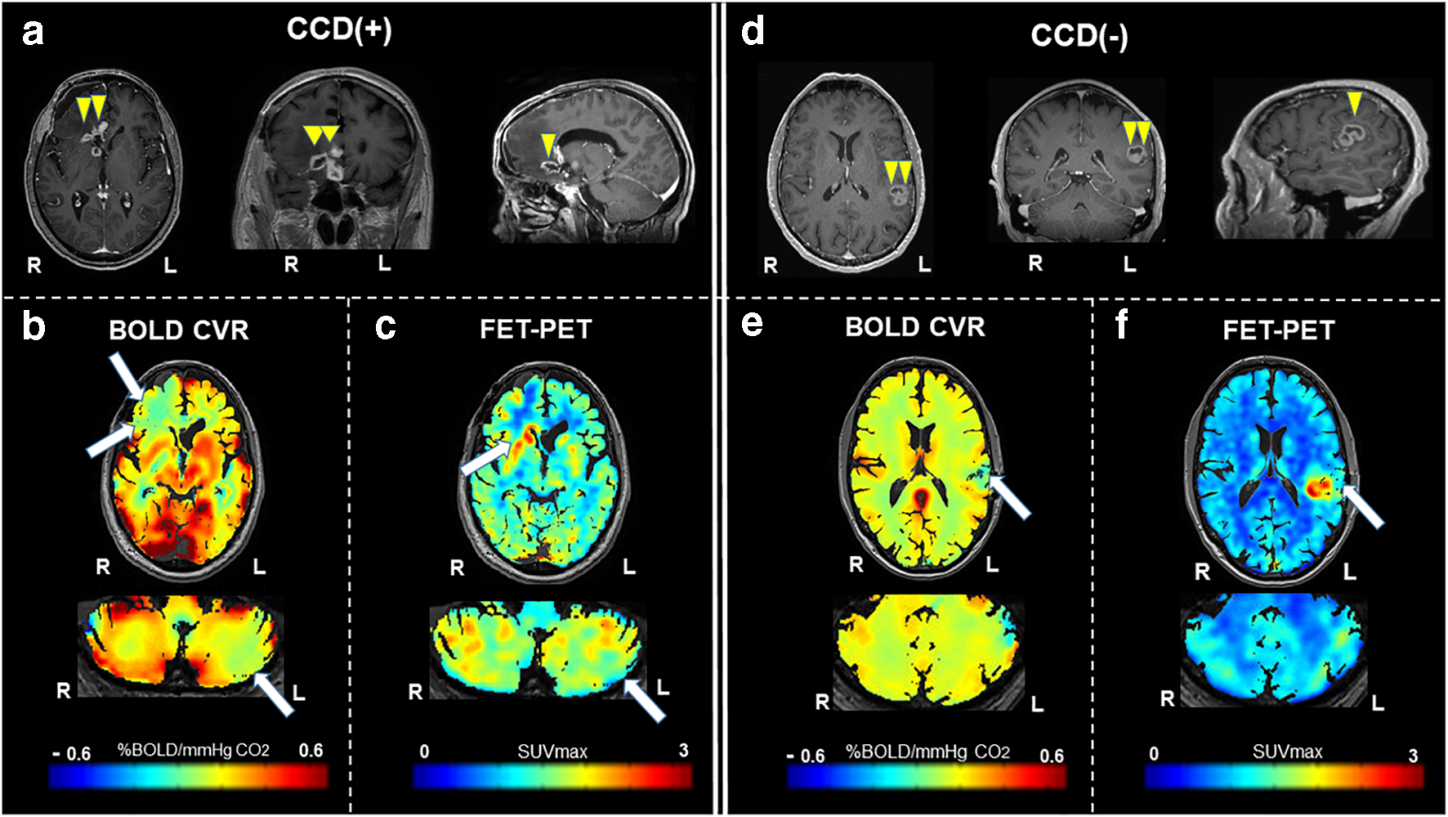
Exemplary images of CCD(+) and CCD(−) patient. (A) A 48-year-old male patients with right-sided frontal glioblastoma (IDH wildtype WHO grade IV), first diagnosed and subtotal resected in August 2017. In February 2018, the patient presented with recurrent tumor frontal on the right side as seen in contrast-enhanced T1-weighted images. He presented with psychomotor slowing down and frontal lobe syndrome (Karnofsky 80%, DRS 2 and mRS 2). He underwent a BOLD-CVR and FET-PET study in a time frame of 12 days. (B) BOLD-CVR showed hemispherical impaired CVR (white arrow) clearly beyond the tumor borders as well as impaired CVR of the contralateral hemisphere. In his cerebellum, cerebellar asymmetry can be appreciated contralateral to the supratentorial tumor recurrence, i.e., crossed cerebellar diaschisis (white arrow on bottom images). (C) FET-PET images confirmed a hypermetabolic recurrent tumor lesion (white arrow). In his cerebellum, crossed cerebellar diaschisis (white arrow on bottom images) is appreciated with good spatial agreement between both imaging modalities. After 3 months, he showed a slight worsening of his condition (Karnofsky 70%, DRS 2 and mRS 2). (D) A 64-year-old male patient with contrast-enhancing tumor on T1-weighted images in the left supramarginal gyrus. He underwent a BOLD-CVR and FET-PET study in a time frame of 23 days. He presented with incomplete angular gyrus syndrome (Karnofsky 80%, DRS 1 and mRS 2). After total resection, the histopathological examination confirmed the diagnosis of glioblastoma (IDH wildtype WHO grade IV). (E) The preoperative BOLD-CVR showed locally impaired CVR (white arrow). No crossed cerebellar diaschisis is observed in BOLD-CVR images. (F) The preoperative FET-PET images confirmed a hypermetabolic tumor lesion (white arrow). Similarly, no crossed cerebellar diaschisis is observed in FET-PET images. After 3 months, he presented with improved overall and neurological condition (Karnofsky 90%, DRS 1 and mRS 1) BOLD, blood oxygenation level–dependent; CCD, crossed cerebellar diaschisis; CVR, cerebrovascular reactivity, defined as percentage BOLD signal change per mmHg CO_2_; DRS, disability rating scale; mRS, modified Rankin scale; FET-PET, O-(2-[18F]fluoroethyl)-L-tyrosine positron emission tomography; SUV, standardized uptake value; WHO, World Health Organization

Using BOLD-CVR as the reference imaging standard [3], five subjects (26.3%) with diffuse gliomas were classified as CCD-positive (CCD(+)). Representative illustrations of one subject each with and without CCD are given in Fig. 2.

The PET CAI showed a difference (3.69 ± 5.09 vs. 1.06 ± 2.35, p = 0.04) between the CCD(+) and CCD(−) groups, indicating, on average, less metabolic activity in the CCD-affected cerebellar hemisphere. The affected (crossed) cerebellar hemisphere exhibited lower CVR values in CCD(+) group (CCD(+) vs. CCD(−): 0.08 ± 0.12 vs. 0.19 ± 0.04, *p* = 0.005) as well as the unaffected (ipsilateral) cerebellar hemisphere did (CCD(+) vs. CCD(−): 0.11 ± 0.12 vs. 0.19 ± 0.05, *p* = 0.04).

### BOLD-CVR Findings in Patients With and Without CCD

For the supratentorial brain region, CCD(+) patients showed significantly more impaired whole-brain CVR (CCD(+) vs. CCD(−): 0.08 ± 0.12 vs. 0.18 ± 0.03, *p* = 0.04) as well as CVR within both the affected (CCD(+) vs. CCD(-): 0.08 ± 0.11 vs. 0.18 ± 0.04, *p* = 0.03) , as well as the unaffected (CCD(+) vs. CCD(−): 0.08 ± 0.12 vs. 0.19 ± 0.04, *p* = 0.04) supratentorial hemisphere as compared with CCD(−) subjects, corrected for tumor grade and primary/recurrence tumor presentation as possible biases (Table 2). These differences were independent of tumor volume (*p* = 0.37).

**Table 2:** CCD according to BOLD-CVR: BOLD-CVR and PET findings

Functional measurement (mean ± standard deviation)	Total cohort (*n* = 19)	Crossed cerebellar diaschisis positive group (*n* = 5)	Crossed cerebellar diaschisis negative group (*n* = 14)	*p*-value
Mean BOLD-CVR whole brain	0.16 ± 0.08	0.08 ± 0.12	0.18 ± 0.03	0.04*
Mean CVR gray matter	0.18 ± 0.08	0.10 ± 0.13	0.21 ± 0.04	0.05*
Mean CVR white matter	0.11 ± 0.07	0.04 ± 0.11	0.13 ± 0.02	0.05*
Mean BOLD-CVR affected supratentorial hemisphere	0.15 ± 0.08	0.08 ± 0.11	0.18 ± 0.04	0.03*
Mean BOLD-CVR unaffected supratentorial hemisphere	0.16 ± 0.08	0.08 ± 0.12	0.19 ± 0.04	0.04*
FET-PET CAI (%)	1.79 ± 3.66	3.69 ± 5.09	1.06 ± 2.35	**0.04**
Tumor volume (cm^3^)	22.33 ± 39.06	36.12 ± 62.04	17.41 ± 28.79	0.37
BOLD-CVR tumor	0.03 ± 0.07	0.01 ± 0.10	0.04 ± 0.05	0.86*
*Using ANCOVA corrected for WHO grade and primary/recurrent tumor as possible covariate *BOLD* blood oxygenation level–dependent; *CAI* cerebellar asymmetry index; *CVR* cerebrovascular reactivity, defined as percentage BOLD signal change per mmHg CO_2_; *FET-PET* O-(2-[18F]fluoroethyl)-L-tyrosine positron emission tomography; *n* number				

### Clinical Performance Status and Outcome

Significant differences between CCD(+) and CCD(−) groups were seen for initial (collected by BOLD-CVR scan) KPS and DRS by the diagnosis of primary diffuse glioma or by first diagnosis of tumor recurrence. This significant difference in both clinical scores persisted at 3-month follow-up (Table 3).

**Table 3:** Clinical performance status and outcome

Clinical outcome (median (interquartile))	Total cohort (*n* = 19)	Crossed cerebellar diaschisis positive group (*n* = 5)	Crossed cerebellar diaschisis negative group (*n* = 14)	*p*-value
KPS scan	90 (10)	80 (20)	90 (10)	0.02*
mRS scan	1 (1)	2 (2)	1 (1)	0.12*
DRS scan	1 (1)	2 (3)	1 (1)	0.03*
KPS 3 months	80 (20)	70 (20)	90 (20)	0.03*
mRS 3 months	2 (1)	2 (1)	1 (1)	0.13*
DRS 3 months	2 (1)	3 (3)	1 (1)	0.01*

*Using ANCOVA corrected for tumor volume as possible covariate

*DRS* disability rating scale, *KPS* Karnofsky performance status, *mRS* modified Rankin scale, *n* number

Table 4 shows the association between BOLD-CVR findings and clinical scores for the whole cohort (19 datasets of 18 patients). Despite tumor volume correction, the BOLD-CVR CAI had a strong positive correlation with all clinical scores by scan (*p*-values for KPS, mRS, and DRS: 0.04, 0.05, 0.02, respectively) and by 3-month follow-up (*p*-values for KPS, mRS, and DRS: 0.03, 0.05, 0.02, respectively), indicating that a larger cerebellar difference was associated with higher scores. Impaired supratentorial BOLD-CVR was still associated with poor initial (by BOLD-CVR scan) clinical performance after tumor volume correction (*p*-values for KPS, mRS, and DRS by scan: 0.01, 0.004, 0.03, respectively).

**Table 4:** Spearman rank correlation for whole cohort (*n* = 19) with partial correction for tumor volume

	KPSscan	mRSscan	DRSscan	KPS (3 months)	mRS (3 months)	DRS (3 months)
BOLD-CVR CAI	**0.04**	**0.05**	**0.02**	**0.03**	**0.05**	**0.02**
BOLD-CVR whole brain	**0.01**	**0.004**	**0.03**	0.54	0.68	0.77

*BOLD* blood oxygenation level–dependent; *CAI* cerebellar asymmetry index; *CVR* cerebrovascular reactivity, defined as percentage BOLD signal change per mmHg CO_2_; *DRS* disability rating scale; *KPS* Karnofsky performance status; *mRS* modified Rankin scale

## Discussion

In our study, the presence of crossed cerebellar diaschisis in patients with diffuse glioma is associated with severely impaired supratentorial cerebrovascular reactivity and worse clinical outcome at 3-month follow-up. By using BOLD-CVR, CCD was diagnosed in 26.3% of subjects, which was confirmed with FET-PET imaging demonstrating marked hypometabolism in the affected crossed cerebellar hemisphere for these patients. Interestingly, the presence of CCD showed a significant BOLD-CVR impairment in both the tumor-affected and tumor-unaffected supratentorial hemispheres, independent of tumor volume and corrected for tumor grade and primary/recurrence tumor presentation as possible biases.

### Meaning of Impaired Supratentorial BOLD-CVR in Diffuse Glioma Patients Exhibiting Crossed Cerebellar Diaschisis

The globally supratentorial impaired BOLD-CVR in the CCD(+) group is a finding in concordance with an earlier study done in cerebrovascular steno-occlusive patients [3], where the impaired BOLD-CVR was now not only seen in the affected supratentorial hemisphere (i.e., the hemisphere harboring glioma) but also in the unaffected hemisphere. In contrast to steno-occlusive disease where after an infarction the trend is generally toward brain repair and functional recovery, diffuse gliomas are dynamic entities which grow, invade, and produce progressive structural and functional deficits [16–18]. Due to their infiltrative nature, the biggest issue in patients with diffuse glioma is tumor border delineation as tumor infiltration outside the contrast enhancement is often unclear. The bilateral BOLD-CVR impairment in CCD(+) patients show that CCD is associated with a more global hemodynamic effect of the tumor on brain tissue outside of its visual tumor borders. This was also found by Liu et al. [2] who showed more widespread perfusion and structural impairments in glioma patients with CCD. A recent paper showed the integration of tumor cells into neural circuits in the brain [19]. Activation of neurons will cause vasodilatation due to physiological neurovascular coupling [20, 21], which will reduce the CVR. In addition, blood flow recruitment to these brain regions will also result in a CVR decrease in other areas [22, 23].

Others have investigated supratentorial structural deficits in glioma patients with CCD and shown that CCD in cerebral gliomas was associated with the pathological grade and lesion size, supporting the concept that diffuse gliomas with more remote effects are more likely to cause CCD [2]. We did not find an increase in lesion size to differ between patients with and without CCD; however, lesion size presented in the prior paper included cerebral edema, making it a broader variable more likely to include infiltration and tumor invasiveness. The glioma grading represents primarily the invasiveness of glioma to infiltrate surrounding tissue. In an earlier paper, we have shown that increasing tumor volume and edema presents with more supratentorial BOLD-CVR impairment [24]. There may, therefore, be a strong interaction between impaired supratentorial BOLD-CVR and the presence of CCD. This finding supports our previously published theory about hemodynamic-induced CCD [3]. As seen in the supplemental file (Table 1), not all diffuse gliomas are anatomically located in the area where the fibers of cortico-ponto-cerebellar tract run, so lesion-induced CCD, as published by Baron [15], cannot be the pathophysiological explanation of (all) our results. Therefore, the overall significantly impaired supratentorial CVR in the CCD(+) group confirms the postulated hemodynamic origin of remote cerebellar impairment in patients with diffuse glioma. Moreover, impaired supratentorial hemodynamic could lead to supratentorial hemispheric atrophy, as already seen in patients with supratentorial disease in whom CCD was suggested by PET [25]. This would support the “hibernating brain” theory as seen in chronic steno-occlusive patients [26].

### Clinical Meaning of Crossed Cerebellar Diaschisis in Diffuse Glioma Patients

The indication of a more widespread effect of the tumor on the brain in patients with CCD is congruent with findings of worse clinical status and a worse prognosis. Already in the 1990s, the presence of CCD was correlated to an increased likelihood of motor impairment [16], a finding which is related to higher NIHSS and functional scores such as the KPS in patients with CCD in our study. Moreover, others investigating the prognostic value of CCD in diffuse glioma patients reported reduced survival in patients with CCD [9]. This strong impact of impaired cerebral hemodynamic on clinical outcome could be (partly) explained by the recently published study, which postulated that tumor cells behave like neurons [19]. This study, for the first time, showed that tumor cells integrate into neural circuits in the brain [19], a finding which can explain why those tumors not only exhibit a locally impaired cerebral hemodynamic but have an impact on whole brain too, as seen in our cohort. With the knowledge that tumor cells integrate into the neural circuit, the impaired supratentorial hemodynamic can be explained. The impaired hemodynamic causes CCD as well as worse neurological status and outcome. Both oncological scores (KPS and DRS) showed clear statistical difference with worse initial status and worse 3-month outcome for CCD(+) group compared with CCD(−) group, even after correction for tumor volume.

This may indicate that CCD could be used as a novel parameter for prognostic models and may have the potential to strengthen future clinical prediction algorithms because of its unique capability to test occult tumor invasiveness. If taken together, the presence of CCD in patients with diffuse glioma may, therefore, be a reflection of the extent of tumor infiltration outside its visible borders as seen in T1-weighted contrast-enhanced scans or on FLAIR and/or T2 sequences and potentially be used in future models as a surrogate imaging marker of tumor invasiveness. Moreover, additional imaging parameters such as perfusion MRI, MR spectroscopy, diffusion-weighted imaging (DWI), and susceptibility-weighted imaging (SWI) should be analyzed to provide additional information about the most aggressively growing parts of diffuse gliomas and their infiltration. To study and evaluate these suspicions, larger studies are needed.

## Limitations

Our data must be interpreted in the context of the study design. First, the study cohort was small (i.e., 19 datasets from 18 patients), but in concordance with other CCD studies in glioma patients [2, 16, 24]. Moreover, we have only included five patients with CCD, and, therefore, the results should be interpreted with caution. However, due to the strong associations found, we believe these results are indicative. Since including 19 datasets from 18 patients, we were able to correct for two variances that could influence our CVR results, we corrected for WHO tumor grade as well as for primary/recurrent tumor and did not correct for previous chemotherapy treatment, which could affect the blood-brain barrier and the tumor hemodynamic. Secondly, this was a retrospective analysis of prospectively collected data stored in a database. Caution should also be taken, regarding the clinical and prognostic validity of a CCD diagnosis as all studies were undertaken with a rather small cohort. Larger studies are necessary to confirm these findings. As the clinical correlation of CCD should be further elucidated, it is also imperative to better understand longitudinal structural and hemodynamic changes within brain remote from the area of primary lesion. Similarly, as seen in other recent BOLD-CVR studies in steno-occlusive disease [3, 9], the cerebral blood flow asymmetry values used to define CCD were based on a region covering a large portion of the cerebellum. Decreased CVR values and hypometabolism are not present for the whole cerebellar hemisphere (as can be seen in Fig. 2B). A more detailed analysis of cerebellar anatomical and functional areas may therefore be considered in future work investigating CCD in diffuse glioma.

## Conclusion

The presence of crossed cerebellar diaschisis in patients with diffuse glioma is associated with impaired supratentorial cerebrovascular reactivity and worse clinical outcome.

## Electronic supplementary material

ESM 1 (DOCX 16 kb)
