# Supplementary material for: Crossed Cerebellar Diaschisis in Patients with Diffuse Glioma Is Associated with Impaired Supratentorial Cerebrovascular Reactivity and Worse Clinical Outcome
Source: Cerebellum. 2020 Jul 31;19(6):824–32. doi: 10.1007/s12311-020-01174-y (PMC7588366; doi:10.1007/s12311-020-01174-y)
Supplement: Supplementary file 1 — (DOCX 16 kb) [file 12311_2020_1174_MOESM1_ESM.docx]

Supplemental file 1: Subject characteristics

| **Case** | **Age**  **(years)** | **Sex** | **Tumor location** | **Histology and tumor**  **WHO-grade** | **Primary/**  **Recurrence** | **CCD(+)/**  **CCD(-)** | **Tumor volume**  **(cm^3^)** | **Cortisone**  **therapy at the time point of imaging** | **Previous**  **chemotherapy**  **(by primary tumor treatment)** |
| --- | --- | --- | --- | --- | --- | --- | --- | --- | --- |
| 1 | 39 | M | Left frontal | Anaplastic astrocytoma  (WHO III) | Recurrence | CCD(-) | 14.76 | No | temozolomide |
| 2* | 53 | M | Right frontal | Anaplastic oligodendroglioma  (WHO III) | Recurrence | CCD(-) | 3.25 | No | PCV |
| 3 | 32 | F | Right temporal | Anaplastic astrocytoma  (WHO III) | Recurrence | CCD(-) | 2.55 | No | none |
| 4 | 63 | M | Left temporal | Glioblastoma  (WHO IV) | Primary | CCD(-) | 4.33 | Yes | / |
| 5 | 37 | M | Right frontal | Anaplastic oligodendroglioma  (WHO III) | Primary | CCD(-) | 105.10 | No | **/** |
| 6 | 45 | M | Right temporo-parietal | Anaplastic oligodendroglioma  (WHO III) | Recurrence | CCD(-) | 40.69 | No | none |
| 7 | 70 | F | Right frontal | Glioblastoma  (WHO IV) | Primary | CCD(-) | 18.78 | Yes | / |
| 8 | 61 | F | Left parietal | Glioblastoma  (WHO IV) | Recurrence | CCD(-) | 0.12 | No | temozolomide, study medication |
| 9 | 52 | M | Left frontal | Anaplastic oligodendroglioma  (WHO III) | Primary | CCD(-) | 5.79 | No | / |
| 10 | 52 | M | Left frontal | Anaplastic oligodendroglioma  (WHO III) | Recurrence | CCD(+) | 0.92 | No | Temozolomide |
| 11 | 54 | M | Left temporo-occipital | Glioblastoma  (WHO IV) | Recurrence | CCD(-) | 5.73 | No | Temozolomide |
| 12 | 60 | M | Right frontal | Anaplastic oligodendroglioma (WHO III) | Recurrence | CCD(-) | 0.80 | No | PCV |
| 13 | 59 | M | Right frontal | Anaplastic oligodendroglioma (WHO III) | Recurrence | CCD(-) | 1.11 | No | Temozolomide |
| 14 | 28 | M | Right frontal | Anaplastic oligodendroglioma (WHO III) | Recurrence | CCD(-) | 0.19 | No | PCV |
| 15 | 49 | M | Right frontal | Glioblastoma  (WHO IV) | Recurrence | CCD(+) | 15.48 | Yes | Temozolomide |
| 16 | 48 | M | Left temporal | Anaplastic oligodendroglioma (WHO III) | Recurrence | CCD(-) | 40.54 | No | Temozolomide |
| 17 | 72 | M | Left multifocal (basal ganglia, hippocampus, mesencephalon) | Glioblastoma  (WHO IV) | Primary | CCD(+) | 7.45 | Yes | / |
| 18 | 68 | M | Right temporal | Glioblastoma  (WHO IV) | Primary | CCD(+) | 10.03 | Yes | / |
| 19* | 51 | M | Right frontal | Anaplastic oligodendroglioma (WHO III) | Primary | CCD(+) | 146.70 | No | / |
| *the patient underwent the protocol two times (initial and by tumor recurrence)  BOLD: blood-oxygenation level dependent , PCV: procarbazine, lomustine and vincristine, PET: Positron Emission Tomography WHO: World Health Organization | | | | | | | | | |
